# Molecular and serological diagnosis of multiple bacterial zoonoses in febrile outpatients in Garissa County, north-eastern Kenya

**DOI:** 10.1038/s41598-024-62714-8

**Published:** 2024-05-28

**Authors:** Martin Wainaina, Johanna F. Lindahl, Anne Mayer-Scholl, Christoph-Martin Ufermann, Jean-Baka Domelevo Entfellner, Uwe Roesler, Kristina Roesel, Delia Grace, Bernard Bett, Sascha Al Dahouk

**Affiliations:** 1 Animal and Human Health Program, International Livestock Research Institute, Nairobi, 00100 Kenya; 2 Department of Veterinary Medicine, Freie Universität Berlin, 14163 Berlin, Germany; 3 Department of Biological Safety, German Federal Institute for Risk Assessment, 12277 Berlin, Germany; 4 Department of Clinical Sciences, Swedish University of Agricultural Sciences, 75007 Uppsala, Sweden; 5 Department of Medical Biochemistry and Microbiology, Uppsala University, 75123 Uppsala, Sweden; 6 Institute for Animal Hygiene and Environmental Health, Freie Universität Berlin, 14163 Berlin, Germany; 7 Food and Markets Department, Natural Resources Institute, University of Greenwich, London, ME130NQ UK; 8 Department of Internal Medicine III, RWTH Aachen University Hospital, 52074 Aachen, Germany; 9 Department 1 – Infectious Diseases, Robert Koch Institute, 13353 Berlin, Germany

**Keywords:** Next-generation sequencing, Risk factors, Bacterial infection, Fever, Infectious-disease diagnostics, Metagenomics

## Abstract

Bacterial zoonoses are diseases caused by bacterial pathogens that can be naturally transmitted between humans and vertebrate animals. They are important causes of non-malarial fevers in Kenya, yet their epidemiology remains unclear. We investigated brucellosis, Q-fever and leptospirosis in the venous blood of 216 malaria-negative febrile patients recruited in two health centres (98 from Ijara and 118 from Sangailu health centres) in Garissa County in north-eastern Kenya. We determined exposure to the three zoonoses using serological (Rose Bengal test for *Brucella* spp., ELISA for *C. burnetti* and microscopic agglutination test for *Leptospira* spp.) and real-time PCR testing and identified risk factors for exposure. We also used non-targeted metagenomic sequencing on nine selected patients to assess the presence of other possible bacterial causes of non-malarial fevers. Considerable PCR positivity was found for *Brucella* (19.4%, 95% confidence intervals [CI] 14.2–25.5) and *Leptospira* spp. (1.7%, 95% CI 0.4–4.9), and high endpoint titres were observed against leptospiral serovar Grippotyphosa from the serological testing. Patients aged 5–17 years old had 4.02 (95% CI 1.18–13.70, *p*-value = 0.03) and 2.42 (95% CI 1.09–5.34, *p*-value = 0.03) times higher odds of infection with *Brucella* spp. and *Coxiella burnetii* than those of ages 35–80. Additionally, patients who sourced water from dams/springs, and other sources (protected wells, boreholes, bottled water, and water pans) had 2.39 (95% CI 1.22–4.68, *p*-value = 0.01) and 2.24 (1.15–4.35, *p*-value = 0.02) times higher odds of exposure to *C. burnetii* than those who used unprotected wells. *Streptococcus* and *Moraxella* spp. were determined using metagenomic sequencing. Brucellosis, leptospirosis, *Streptococcus* and *Moraxella* infections are potentially important causes of non-malarial fevers in Garissa. This knowledge can guide routine diagnosis, thus helping lower the disease burden and ensure better health outcomes, especially in younger populations.

## Introduction

Kenya has had remarkable success in the reduction of overall morbidity and mortality, and there has been a marked increase in healthy life expectancy from 54.1 years in 1990 to 58.6 years in 2016. This is largely due to the introduction, and scaling-up from the early 2000s, of many health interventions such as the treatment and control of HIV/AIDS and malaria, child immunisations, and free maternal healthcare^1^. Despite this, communicable diseases are responsible for a large proportion of disability-adjusted life years (DALYs—an aggregate measure of overall health loss that incorporates morbidity and mortality) in the country^1^. Due to the large number of agents that can cause these illnesses, metagenomics can play a role in detecting pathogens in a non-targeted manner in clinical samples when the clinical presentation is ambiguous^2^. Unlike traditional microbiological methods, metagenomic sequencing can detect multiple pathogens present in a clinical sample, enables the detection of unculturable agents, and can be used even after patients have started antibiotic treatment^2^. Consequently, clinical metagenomics has proven useful in several contexts^3,4^. Metagenomics allows for the profiling of the microbial community in a sample by sequencing the DNA using next-generation sequencing methods. When compared to 16S rRNA metagenomics, shotgun metagenomics can determine the bacterial profile of samples even when their 16S rRNA sequences are not well described, thereby enabling the discovery of novel species. The approach can also provide functional insights by illustrating functional genes, including those that confer antimicrobial resistance (AMR), is more sensitive and can offer resolution beyond bacterial genera and species such as strain, and identifies non-bacterial microorganisms such as viruses and protists. This untargeted approach can be quite useful, especially in East Africa where non-malarial fevers are caused by a wide array of bacterial pathogens^5,6^.

There are still huge regional disparities in health outcomes in Kenya. Due to historical inequitable allocation of resources, poverty and insecurity, Kenya’s expansive arid and semi-arid (ASAL) areas have poor coverage of treatment and control programs for important infections. For instance, ownership of insecticide-treated bed nets is lowest in these counties, thereby posing serious challenges in malaria control^7^. In addition, the high prevalence of non-malarial fevers in the region complicates malaria control because most of these illnesses have similar clinical presentations. Brucellosis, Q-fever and leptospirosis are important but often under-diagnosed non-malarial febrile illnesses in ASAL Kenya^8–10^. Brucellosis, Q-fever and leptospirosis are caused by pathogenic bacteria of the genera *Brucella*, *Coxiella* and *Leptospira*, respectively. Fever, headaches, sweating, chills, and myalgia are common clinical presentations in acute illness which are also seen in malaria, which makes differential diagnosis challenging^11–13^. Exposure to these zoonoses is commonly ascertained by serological testing for antibodies against the bacteria to demonstrate acute or chronic courses. The Rose Bengal test is the most widely used screening test for brucellosis and is very helpful in diagnosing human brucellosis in endemic countries. The direct detection of brucellae in clinical samples can also be done using PCRs targeting various loci such as genus-specific insertion sequences (IS*711*, IS*650*), *bscp31*, and *omp 2a*. The reference serological test for Q fever diagnosis is the indirect immunofluorescence assay (IFA), but ELISA testing is recommended for use due to commercial availability, standardised interpretation, easy automation, and good diagnostic performance. There are several PCR assays for Q fever diagnosis, but IS*1111* is highly sensitive due to its several copies, 20 copies of the repetitive element being observed in the *C. burnetii* Nine Mile reference strain for instance. Serological diagnosis is indispensable in demonstrating leptospirosis infections, with the gold standard test being the microscopic agglutination test (MAT). Additionally, direct detection can be done using culture from clinical samples or testing of clinical samples using PCR. Several PCR assays targeting genes of pathogenic leptospires such as *lipL32*, *lig*, and *lfb1* are useful in proving leptospirosis cases with high sensitivity^14–16^.

We therefore undertook a study in Garissa County in ASAL Kenya to estimate the prevalence of brucellosis, leptospirosis and Q-fever in febrile patients (otherwise termed proportional morbidity rates [PMr] when determined in a sick population) and to identify risk factors for exposure to these pathogens. We also used metagenomic sequencing for a non-targeted detection of other important bacterial causes of non-malarial fevers in selected patients. These findings will lead to improved management of febrile illnesses for patients with a malaria-negative test result.

## Results

### Epidemiological and serological results

We recruited 216 febrile patients, 118 from Sangailu and 98 from Ijara health centre. Most were female (n = 150), and the median age of patients was 25 years (interquartile range [IQR] 18–35). Most patients never attended school (n = 148), had no source of income (n = 167) and drew water from unprotected wells (n = 93). The median distance to the healthcare facility for the patients was 7 km (IQR 2–13). The highest PMr estimates observed from the serological testing were those of *C. burnetii* (45.8%, 95% confidence intervals [CI] 39.1–52.7), and fewer patients were exposed to *Brucella* spp. (13.9%, 95% CI 9.6–19.2) and *Leptospira* spp. (3.7%, 95% CI 1.6–7.2). The distribution of the PMr estimates with the various demographic characteristics and putative risk factors is given in supplementary table 1.

According to the MAT results, the highest agglutinating titres were observed in serovar Grippotyphosa (n = 4), with endpoint titres of 1:800, 1:800, 1:1600, and 1:12800. In addition, serovars Bataviae and Tarassovi showed agglutination in one patient and both had endpoint titres of 1:200. The remaining three patients showed agglutination for serovars Icterohaemorrhagiae (1:100), Pyrogenes (1:200), and Sejroe (1:400).

### Risk factors for seropositivity

Results from the univariable logistic regression model for *Brucella* spp. exposure showed income source, water source, age categories, camel herding, disposal of carcasses, sleeping outside with herds, assisting with birthing livestock, disposal of aborted foetuses, and taking care of sick animals as significant predictors (*p*-value < 0.2). However, only age remained a significant predictor at the multivariable level (Table 1). The final logistic regression model showed that patients aged 5–17 had 4.02 (95% CI 1.18–13.70, p-value 0.03) times higher odds of exposure than those aged 35–80 years. Patients who drew water from dams and springs had 1.09 times higher odds of exposure to *Brucella* spp. than those who did so from unprotected wells, although this observation was not statistically significant (*p*-value = 0.07).

**Table 1:** Results of the final multivariable logistic regression models to determine risk factors for exposure to the selected bacterial zoonoses.

Pathogen	Variable	Categories	Total	OR (95% CI)	SE	Z-value	*P*-value
*Brucella* spp.	Age categories	35 to 80	49	Ref			
		25 to 34	45	2.47 (0.71–8.62)	0.64	1.42	0.16
		18 to 24	63	2.22 (0.58–8.48)	0.68	1.17	0.24
		5 to 17	59	4.02 (1.18–13.70)	0.63	2.22	**0.03**
	Water source	Dams and springs	61	Ref			
		Unprotected wells	93	0.43 (0.17–1.08)	0.47	− 1.79	0.07
		Others *	62	0.47 (0.17–1.30)	0.52	− 1.45	0.15
*Coxiella burnetii*	Age categories	35 to 80	49	Ref			
		25 to 34	45	1.11 (0.53–2.31)	0.38	0.27	0.79
		18 to 24	63	1.12 (0.50–2.50)	0.41	0.28	0.78
		5 to 17	59	2.42 (1.09–5.34)	0.40	2.18	**0.03**
	Water source	Unprotected wells	93	Ref			
		Dams and springs	61	2.39 (1.22–4.68)	0.34	2.53	**0.01**
		Others*	62	2.24 (1.15–4.36)	0.34	2.36	**0.02**

OR, Odds ratio; CI, Confidence interval; SE, Standard error; Ref, Reference category.

*Category comprised protected wells, boreholes, bottled water, and water pans.

Significant values are in bold.

The univariable logistic regression models for *C. burnetii* showed several variables with *p*-values < 0.2, but the final model demonstrated that patients aged 5–17 had 2.42 (95% CI 1.09–5.34) times higher odds of exposure to *C. burnetii* than those aged 35–80 (*p*-value = 0.03). In addition, patients who sourced water from dams/springs, and other sources (protected wells, boreholes, bottled water, and water pans), had 2.39 (95% CI 1.22–4.68, *p*-value = 0.01) and 2.24 (1.15–4.35, *p*-value = 0.02) times higher odds of exposure than those who used unprotected wells. Leptospires were excluded from these analyses. Results from the goodness of fit are given in the supplementary information.

### PCR results

PCR positivity was highest for *Brucella* spp., with 19.4% (95% CI 14.2–25.5) patients testing positive. This was followed by *Leptospira* spp., with 1.7% (95% CI 0.4–4.9), and *C. burnetii* (< 1.0%, 95% CI 0.0–1.5%). All patients testing positive for *Brucella* were also confirmed to be positive for *B. melitensis* using the species-specific assay.

### Clinical signs and symptoms

The clinical signs and symptoms of patients positive for *Brucella* by serology and PCR varied (Supplementary Fig. 2). However, most patients exhibited minor splenomegaly, had marginally higher median diastolic and lower median systolic pressures, and a higher respiratory rate.

*Coxiella*-seropositive patients had even more diverse clinical manifestations, with minor splenomegaly, no or moderate headaches, severe seizures, moderate rashes, and moderate presence of blood in stool or vomit, and higher median diastolic and marginally lower systolic pressures (Supplementary Fig. 2).

There were no consistent symptoms in *Leptospira*-positive patients, except for slight elevations of the median diastolic and systolic pressures, and respiratory rates (Supplementary Fig. 2).

### Metagenomic results

**Figure 1:**
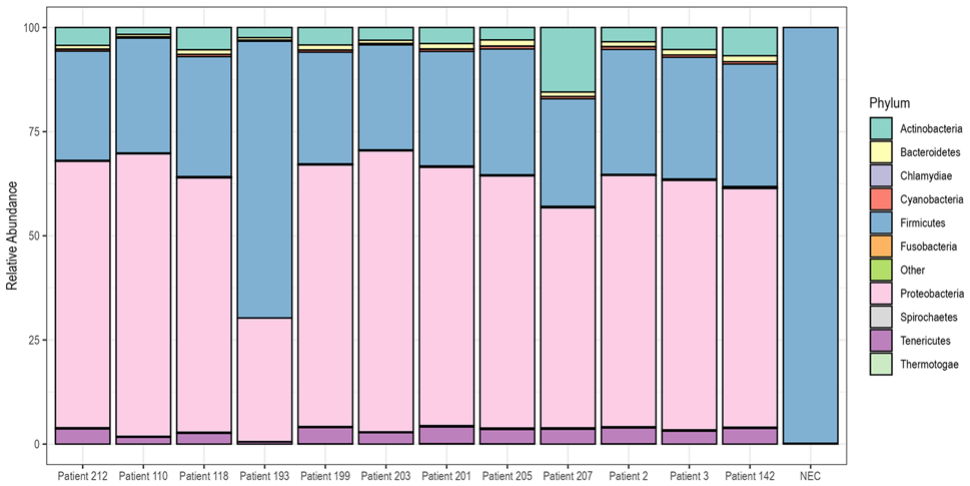
Comparison of bacterial community compositions in patient samples based on metagenomic sequencing. The relative abundance of each bacterial phylum is shown, as identified using *kraken2* and further re-estimated using a Bayesian approach with *bracken*. The data are presented as percentages of the total bacterial community in each sample and “Other” comprised bacteria that had less than 5% relative abundance.

**Figure 2:**
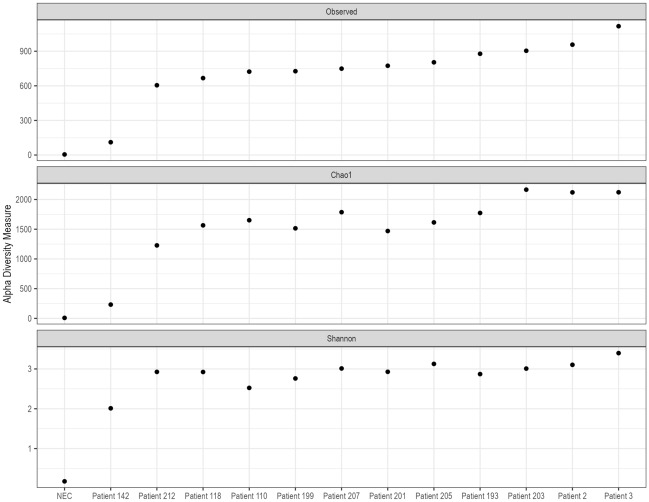
Alpha diversity scores of the metagenomic profiles, calculated using the observed, Chao1, and Shannon indices. These indices represent a measure of species richness and evenness in the datasets, which were obtained through shotgun metagenomic sequencing of patient sera.

**Figure 3:**
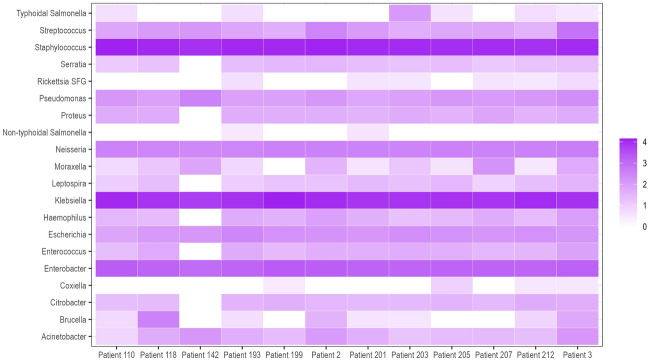
Classified genus- and species-specific reads per million total reads in the metagenomic analyses. To enhance the visualisation and avoid computing log_10_ of zero, a pseudo count of 1 was added to the original data and the transformed values were expressed as log_10_. Therefore, 4 on the heat map scale should be interpreted as 3 in the original data for instance (or 1000 reads per 1,000,000 total reads).

**Figure 4:**
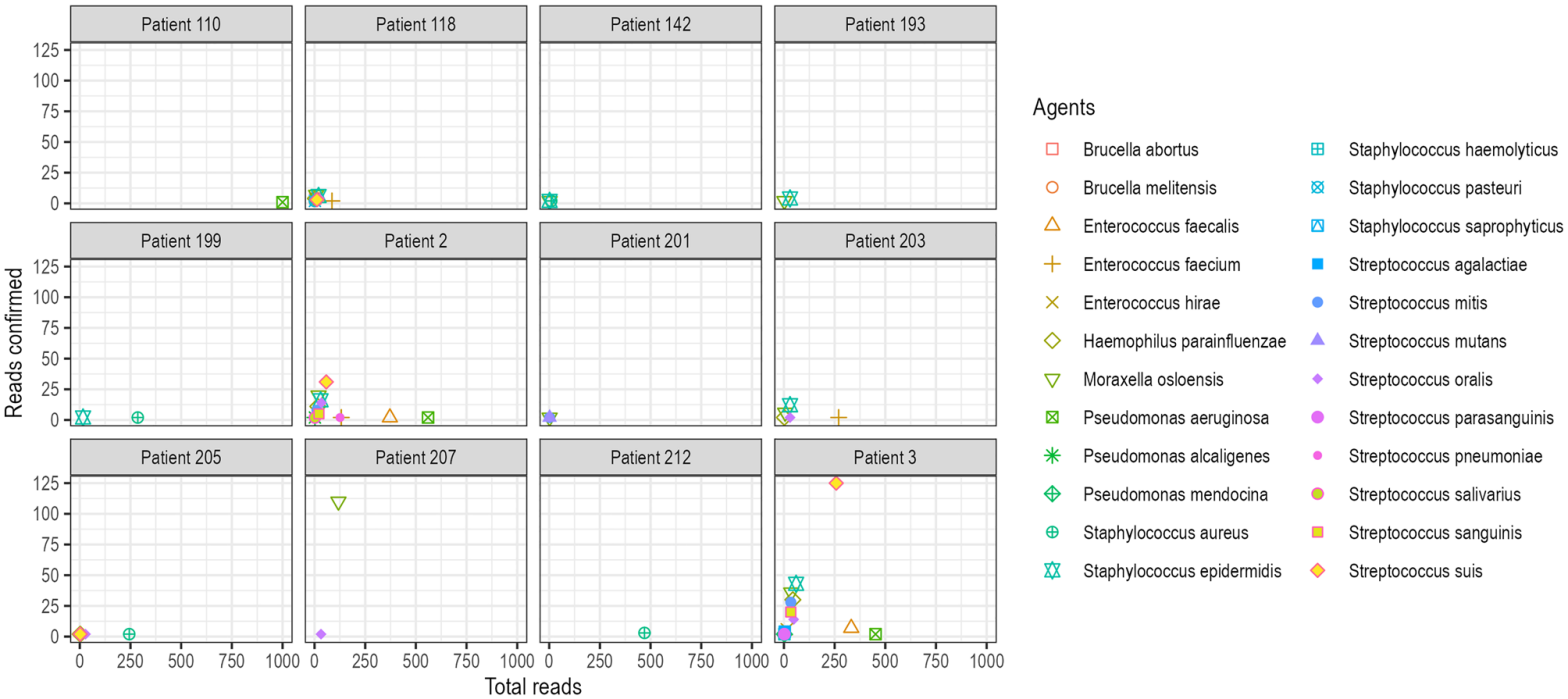
A scatter plot summarizing the number of BLASTn-confirmed reads for the top twenty fever-causing agents in the metagenomic datasets. Each dot represents one agent and its coordinates are the number of reads confirmed against the number of total reads BLAST’ed. The x-axis is capped at 1000 reads to show only the agents that reached the cap, while the y-axis shows the number of confirmed reads. The plot suggests that many agents were confirmed by a low number of reads, and highlights the agents that were confirmed by the most reads.

We obtained a median of 783,410 (IQR 732,804–947,650) pair-end reads using the metagenomic sequencing after quality trimming (Supplementary table 2). The peak insert size was 149 bases. Various bacteria were detected, most from the phyla Firmicutes, Proteobacteria and Tenericutes (Fig. 1). The negative extraction control (NEC) had the lowest alpha diversity scores (observed, Chao1 and Shannon), and samples showed varying alpha diversities, with patient 142 having the lowest and patient 3 having the highest observed score (Fig. 2). There were also reads from the top twenty commonest causes of non-malarial fevers identified, with the most abundant from the metagenomic analyses being *Staphylococcus* and *Klebsiella* spp (Fig. 3). We confirmed 500 random reads taxonomically assigned to the top 20 agents using BLASTn to increase diagnostic specificity. Agents with fewer than 500 had their entire taxonomically assigned reads blasted for confirmation. The most confirmed reads were those of *Streptococcus* spp., with no reads from these 20 agents being detected in the negative control (Fig. 4). A high number of reads allocated to *Moraxella* were also confirmed from the BLASTn analyses in patient 207. AMR genes were found in the following patients; patient 110 (*lmo0919_fam*, coverage = 38.3, depth = 0.9), patient 203 (*qacC*, coverage = 38.3, depth = 1.6), patient 2 (*fosX*, coverage = 43.5, depth = 1.71), patient 3 (*fosX*, coverage = 52.2, depth = 1.0), and patient 205 (*fosX*, coverage = 40.1, depth = 1.6). There were no virulence factors detected.

## Discussion

We investigated bacterial zoonoses (brucellosis, Q-fever, and leptospirosis) in 216 febrile patients who visited two health centres in Garissa County using both targeted and untargeted metagenomic detection methods. High PCR positivity was observed for *Brucella*. High endpoint titres were observed against leptospiral serovar Grippotyphosa. Young patients had higher odds of seropositivity to *Brucella* spp. and *C. burnetii* than their older counterparts. Lastly, AMR genes and other bacteria such as *Streptococcus* and *Moraxella* were found using metagenomic sequencing. Results of this survey reveal multiple causes of non-malarial fevers in Garissa County. This calls for considering brucellosis, leptospirosis and infections from *Streptococcus* and *Moraxella* in routine diagnosis of bloodstream infections to lessen the burden of non-malarial fevers in the region.

Brucellosis is an important cause of non-malarial fevers in Kenya and prevalence at the community level is high. The PMr in our study of 13.9% was comparable to other studies of febrile patients in the region^17^. The prevalence of animal brucellosis is considerably lower than that of humans in the area^18^, and households with one seropositive animal have been shown to have higher odds of having a seropositive human^19^. The monitoring of animal brucellosis through One Health surveillance and control strategies is therefore necessary for lowering the human brucellosis prevalence because exposure is usually from livestock and animal products.

A previous study on febrile patients in the region showed 19.1% exposure by IgG antibodies against *C. burnetii*, an estimate much lower than that in our study^20^. However, cross-reactivity can occur when patients have been exposed to *Bartonella* spp., *Legionella* spp., or *Chlamydia* spp.^21,22^, thereby overestimating the seroprevalence.

Higher leptospirosis PMr has been observed in febrile patients in Kenya^23^, and 3/12 (25%) patients were *Leptospira* IgM positive by ELISA in a 2005 outbreak of acute febrile illness in the region^8^. The exposure of 3.7% of febrile patients therefore demonstrates that leptospirosis should be considered in the differential diagnosis of acute non-malarial fevers in the region.

Our study also determined that younger patients (5–17 years) had higher seroprevalence estimates and were at higher odds of being exposed to *Brucella* and *C. burnetii* than their older counterparts between 35 and 80 years. This may be because younger people come into contact with infected animal hosts more regularly than those aged 35–80, through any activities in the production, slaughter, processing, and retail of animal source foods. These professional activities and consumption habits also have gender-specific considerations which may play a role in this risk factor^24^. Healthcare-seeking behaviour is also higher in younger people with febrile illnesses in Kenya than in older patients^25^, and our observation could therefore have arisen from selection bias. However, community exposure in the country is higher in older patients^19^.

Patients that sourced water from unprotected wells had lower odds of exposure to *C. burnetii*, and tended towards significantly lower odds of exposure to brucellae than those getting water from dams, springs, and other sources. Even though exposure to *C. burnetii* through contaminated environmental water has been documented, it is an uncommon transmission pathway and in need of further elucidation^26^. Contamination of water sources with animal waste can lead to the spread of *C. burnetii*^27^. Brucellae can contaminate water sources when animal waste pollutes water sources, and the bacteria can survive for long periods in water, thereby also posing a risk of human exposure^28,29^. Therefore, the protection of water sources from possible contamination is vital in reducing human exposure to zoonoses. It is also possible that water source could be a surrogate for other factors that facilitate transmission of these infections and were not captured by our questionnaires.

Our study also showed a seroprevalence of 3.7% for leptospires in febrile patients. This is lower than estimates observed in febrile patients in neighbouring Tanzania^30^. Serovars Pyrogenes, Sejroe, Bataviae, Tarassovi and Icterohaemorrhagiae were found in the patients, and Grippotyphosa showed particularly high endpoint titres. Even though high titres against serovars other than the infective one (paradoxical reactions) can happen in acute leptospirosis, these are likely important serovars responsible for human leptospirosis in Garissa and MAT panels should include them. Leptospires were excluded from the risk factor analyses due to the few positives observed, which led to insufficient statistical power to detect any significant effects.

Reads for *Streptococcus* were confirmed by BLASTn analyses in the metagenomic sequencing and these were also not observed in the negative control used, implying this could be a cause of non-malarial fevers in some patients. Similarly, a high number of *Moraxella* reads were confirmed by BLASTn in a patient. Both of these genera are important causes of respiratory tract infections and can be found in invasive infections^31^. The metagenomic datasets generated have the potential to reveal AMR genes which are clinically relevant and cannot be obtained with 16S rRNA amplicon sequencing. There may have been DNA degradation resulting from sample transportation from the remote study sites, and long storage of the archived samples, as evidenced by the short average fragment lengths. Additionally, results from shotgun metagenomic experiments can be confounded by external contaminants at various stages of the workflows. These can originate from extraction and library prep reagents^32^ and various bacteria have been identified as common contaminants^33,34^. The effect of these contaminants is more pronounced in low biomass samples such as serum, as the sample is inundated by contaminating nucleic acid due to the low amount of starting material from the patient, consequently generating misrepresentative results^33^. In addition, index hopping and cross-contamination by carry-over of amplicons from previous runs are sources of internal contamination^32^ and can bias metagenomics studies. Even though some reads were found in our NEC, they could not be assigned to the top 20 leading fever agents, validating our results as free from internal contamination. We also extracted circulating cell-free DNA which according to the manufacturer may not be detected with spectrophotometric methods such as the Qubit™ assay. There is also growing consensus that a blood biome exists for healthy individuals and may be constituted largely by bacteria speculated to be transient and from sources such as the gastrointestinal tract, skin and oral microbiomes. The putative blood biome is largely constituted by *Proteobacteria*, *Actinobacteria*, *Firmicutes*, and *Bacteroidetes*^35^ which were also the largest bacterial phyla found in this study. Therefore, the identification of these bacteria does not offer definitive proof that they are the causative agents of non-malarial fevers. Lastly, a few important AMR genes were determined in some patients. The gene coverage and coverage depth for most was not high, and these results could have originated from low abundance^36^. We therefore cannot establish whether these genes were clinically relevant. However, studies utilising higher sequencing depth could add value in monitoring AMR in febrile patients using metagenomic sequencing.

Clinical signs and symptoms for the different pathogens were not distinctive, with a selected few being associated with positive patients using both serology and PCR tests. Future studies making use of the gold standard diagnostic tests can therefore determine the positive predictive values of these clinical signs and symptoms to aid physicians in the region in the diagnosis of these non-malarial fevers. Most of the *Brucella* isolated from humans in Kenya has been *B. melitensis*^10^, as is the case in other countries^37^, a likely result of *B. melitensis* being more prevalent than *B. abortus*. *B. melitensis* is often considered more pathogenic than *B. abortus* due to its higher association with human brucellosis. However, human brucellosis resulting from *B. abortus* can be equally severe^38^, and preventative measures that lower exposure from cattle, sheep and goats should be adopted in the country.

Our study had several limitations. We utilised shallow metagenomic sequencing due to limited resources which may lead to decreased sensitivity in detecting low concentrations of circulating DNA in clinical samples. Our study also had a relatively small sample size of inpatients and this can lead to failure to detect significant effects of putative risk factors in causing disease exposure. We also did not focus on viral agents due to lack of resources, despite their importance in causing non-malarial febrile illnesses in Kenya. Lastly, the use of archived samples from 2014 means the findings do not necessarily reflect the current disease dynamics.

In conclusion, Garissa County is an ASAL area with a high poverty index. As all patients recruited in this study were malaria-negative, investments in the diagnosis and treatment of zoonotic diseases are key in lowering disease burden, improving health outcomes and increasing the productivity of human capital. Prevention of exposure through occupational risks or consumption of contaminated foods, especially to younger individuals, should be considered to lower the high exposures observed. The presence of multiple zoonotic pathogens in febrile patients presenting in local health centres shows the urgent need for surveillance and control programs in the county. Using non-targeted sequencing approaches can add value in detecting uncommon causes of febrile illnesses, especially when recently collected samples are obtained and appropriate contamination controls are included in the sequencing process. Lowering the burden of these diseases in animal hosts is cost-effective. Investments in the local health centres for routine screening of these zoonoses, and sensitisation of medical professionals on the importance of non-malarial fevers in the region should also be carried out to ensure proper use of anti-malarial and antibiotic therapies. Protection of water sources from animal waste should also be encouraged to minimise zoonotic disease exposure.

## Methods

### Study site

Garissa County is located in the eastern region of Kenya and borders Wajir, Isiolo, Tana River, Lamu counties, and the country of Somalia. The county is an ASAL region and has some of the highest poverty rates in the country^39^. There are seven Sub-counties in Garissa which comprise Balambala, Dadaab, Fafi, Garissa, Hulugho, Ijara, and Lagdera. The main economic activity of the region is pastoral farming.

### Study patients and inclusion criteria

Patients seeking treatment for febrile illnesses were recruited and blood samples were obtained according to a protocol approved by the Ethics and Scientific Review Committee of the Africa Medical and Research Foundation (Approval number REF: AMREF-ESRC P65/2013). Only participants above five years old were included and informed consent was sought from all participants (or their guardians for minors) and all methods in this study done according to the approved ethical guidelines and regulations. We collected blood samples from 216 febrile patients from Ijara (98 patients) and Sangailu (118 patients) health centres in Garissa County of Kenya (Supplementary Fig. 1) from March to September 2014. This was part of a larger study on dynamic drivers of diseases exploring the influence of land use changes on Rift Valley fever, and other important emerging infectious diseases were investigated concurrently. The sample size was estimated using the formular for designing prevalence studies^40,41^. An a priori prevalence of Rift Valley fever was assumed to be 20%^42,43^.

The study was designed to estimate this prevalence with a confidence of 95% and a reliability of 5%. Based on these assumptions, an optimal sample size of 245 was derived. The study, however, obtained 216 subjects. Pretested questionnaires uploaded to smartphones using the open data kit tool were administered to the participants in Somali and Swahili by clinical officers as they awaited services. The questionnaire data collected included: location, gender, occupation, age, and level of education. The potential risk factors for exposure to important and common non-malarial febrile illnesses were derived from the literature and covered topics such as the source of water used, livestock ownership, contact with livestock, and ease of access to public health services. We considered only malaria-negative patients to avoid confounding results with malarial infections which have a similar clinical presentation.

### Laboratory analyses

#### Serological and PCR testing

Blood in plain vacutainers was centrifuged at 1500 × *g* for 20 min to obtain serum. Testing for malaria was done using the CareStart™ Malaria Pf (HRP2) Ag rapid diagnostic test (Access Bio, Inc.) according to the manufacturer’s instructions. The test is highly sensitive in detecting the histidine-rich protein II (HRP-II) antigen specific to *P. falciparum* and is approved for diagnosis of *P. falciparum* malaria which is responsible for 99% of malaria cases in Kenya^7,44^. Serological testing for the three bacterial infections was performed as follows: the Rose Bengal test (Pourquier® Rose Bengale Ag, IDEXX) for *Brucella* spp., the *Coxiella burnetii* Phase 2 IgG and IgM ELISA (Institut Virion\Serion GmbH) for *C. burnetii*, and lastly, the MAT test for *Leptospira* spp. We included the following 12 leptospiral reference strains which are relevant in Kenya: Ballico, Akiyami A, Mus 127, Swart, Hond Utrecht IV, Moskva V, RGA, Hebdomadis, Pomona, Salinem, M 84, and Perepelitsin. Samples with MAT titres ≥ 1:100 were regarded as positive and were further tested to 1:12,800 to determine endpoint titres.

DNA was isolated from heparinised blood and tested using PCR as previously done in a related study^18^. This involved testing for *Brucella* spp. by targeting both the *bcsp31* and IS*711* loci, and further testing positive samples for *B. melitensis* and *B. abortus* with the species-specific IS*711* assays. PCRs for pathogenic leptospires and *C. burnetii* targeted *lipL32* and IS*1111*, respectively.

#### Next-generation sequencing

We randomly selected a subset of nine patient samples for these analyses. This allowed us distribute the maximum sequencing output capacity of 39Gb across the samples, thereby enabling adequate sequencing depth. Circulating cell-free DNA was isolated from serum samples using the QIAamp minElute ccfDNA mini kit (Qiagen), and included nuclease-free water during DNA extraction as the NEC. Sequence libraries for the DNA were prepared using the Nextera DNA flex library prep kit (Illumina) and sequencing was performed on an Illumina NextSeq 500 platform with a 150-cycle paired-end configuration.

### Bioinformatic analyses

Trimming and quality checks of sequence reads were done with *fastp* version 0.20.1, and a mean phred score of 30 or above (Q30) was used to filter low-quality reads. Human reads were further filtered out from trimmed reads using *bowtie2* version 2.3.5.1. Taxonomic identification and abundance estimation was performed with *kraken2* version 2.1.2, and later re-estimated with *bracken* version 2.5, using the RefSeq release 95 database. Pathogenic species were filtered as per the guidelines of the American Biological Safety Association^45^, and a subset not exceeding 500 reads taxonomically assigned to these pathogenic species was extracted and confirmed using BLASTn as detailed by Gruetzke et al.^36^. We reported any agents comprising the twenty leading causes of non-malarial fevers in eastern Africa as determined by Wainaina et al.^6^. We determined the presence of virulence factors and AMR genes by SRST2 version 0.2.0 (minimum coverage = 30% and minimum depth = 1) using the Virulence Factor Database (VFDB set A_nt downloaded on March 22, 2018) and National Center for Biotechnology Information (NCBI) AMRfinder database version 2019-10-30.1, respectively^36^. Plots were created in R statistical environment using *phyloseq* version 1.38.0, *patchwork* version 1.1.2, *pheatmap* version 1.0.12 and *ggplot2* version 3.4.0.

### Epidemiological analyses

Data analysis was done on R. We initially determined the distribution of PMr estimates for *Brucella* spp., *C. burnetii* and *Leptospira* spp. with the patient metadata. Univariable logistic regression models were fitted with healthcare facilities as random effects. The variables with *p* < 0.2 were included in the multivariable models and variables were removed using backward elimination until there was no evidence of confounding. Significant association was deemed when *p* < 0.05. The generalized linear mixed models were fitted using *lme4* version 1.1–31. We also determined the distribution of clinical signs and symptoms of patients that were sero- and PCR-positive. Further details on these analyses are given in the supplementary information.

### Ethical approval

Ethical approval was given by the Ethics and Scientific Review Committee of the Africa Medical and Research Foundation (Approval number REF: AMREF-ESRC P65/2013). Only participants above five years old were recruited and informed consent was sought from all participants (or their guardians for minors). Informed consent was obtained from legally authorized representatives for participants who could not read and write.

### Supplementary Information

Supplementary Information.

## Data Availability

Raw dehumanised reads generated by next-generation sequencing have been uploaded to the European Nucleotide Archive (ENA) under project accession PRJEB65963 (https://www.ebi.ac.uk/ena/browser/view/PRJEB65963). The datasets generated during and/or analysed during the current study are available from the corresponding author on reasonable request.
