# Supplementary material for: Molecular and serological diagnosis of multiple bacterial zoonoses in febrile outpatients in Garissa County, north-eastern Kenya
Source: Sci Rep. 2024 May 28;14:12263. doi: 10.1038/s41598-024-62714-8 (PMC11133362; doi:10.1038/s41598-024-62714-8)
Supplement: Supplementary file 1 — Supplementary Information. [file 41598_2024_62714_MOESM1_ESM.pdf]

Supplementary information:

Molecular and serological diagnosis of multiple bacterial zoonoses in febrile outpatients in Garissa County, north-eastern Kenya

Martin Wainaina, Johanna F. Lindahl, Anne Mayer-Scholl, Christoph-Martin Ufermann, Jean-Baka Domelevo Entfellner, Uwe Roesler, Kristina Roesel, Delia Grace, Bernard Bett, Sascha Al Dahouk

Epidemiological data analyses

Patient metadata was merged with diagnostic test results on R statistical environment version 4.1.1. The patients' ages were converted to a categorical variable at the 25th and 75th percentiles, and distance to the healthcare facility was divided at the median into two categories. Both continuous variables did not follow a normal distribution when assessed by the Shapiro-Wilk test and visually inspected using Q-Q plots and histograms, even after applying square root and log transformations. A distribution of PMr estimates for *Brucella* spp., *C. burnetii* and *Leptospira* spp. was determined for the variables created from the patient metadata. We then assessed risk factors for exposure by fitting logistic regression models. We initially first tested for unconditional associations using the chi-square test as an initial exploratory analysis of the variables, and checked for correlation of variables included in the models to avoid multi-collinearity ($p<0.05$), as this would make the models unstable. We eliminated variables that did not have much variation between their categories (e.g., livestock enclosed). We fitted univariable models for *Brucella* and *C. burnetii*, and incorporated healthcare facilities as random effects to account for any potential clustering effects from the sampling sites. The variables with $p<0.2$ in the univariable analyses were added to the multivariable models. We eliminated variables from the model using backward elimination if there was no evidence of confounding in order to identify potential predictors for further investigation. Therefore, only variables that demonstrated a significant

association with the outcome ($p<0.05$), even after adjusting for other variables, were included in the final model. The generalized linear mixed models were fitted using *lme4* version 1.1-31. Leptospires were excluded from these analyses due to the few positives observed. We assessed whether the final models used in the study were different from null models (with intercept only) by comparing the two using the likelihood ratio test and checking for the differences in the deviances and Akaike information criterion (AIC) estimates of the two models. We lastly determined the distribution of clinical signs and symptoms of patients that were sero- and PCR-positive. For this, arcsine transformed proportions of positive patients (to stabilise the variance and enhance the visualisation) were represented in a heat map for the categorical variables, and those of continuous variables as a panel of boxplots using *ggplot2* version 3.4.0.

Goodness of fit for models

Results from the likelihood ratio test comparing the final *Brucella* model with a null model showed that the final model had lower AIC and residual deviances (p-value = 0.09). The comparison also showed lower AIC estimates and residual deviances for the final *C. burnetii* model (p-value =0.02), thus demonstrating that the final models used in our study had a good fit.

Supplementary figures

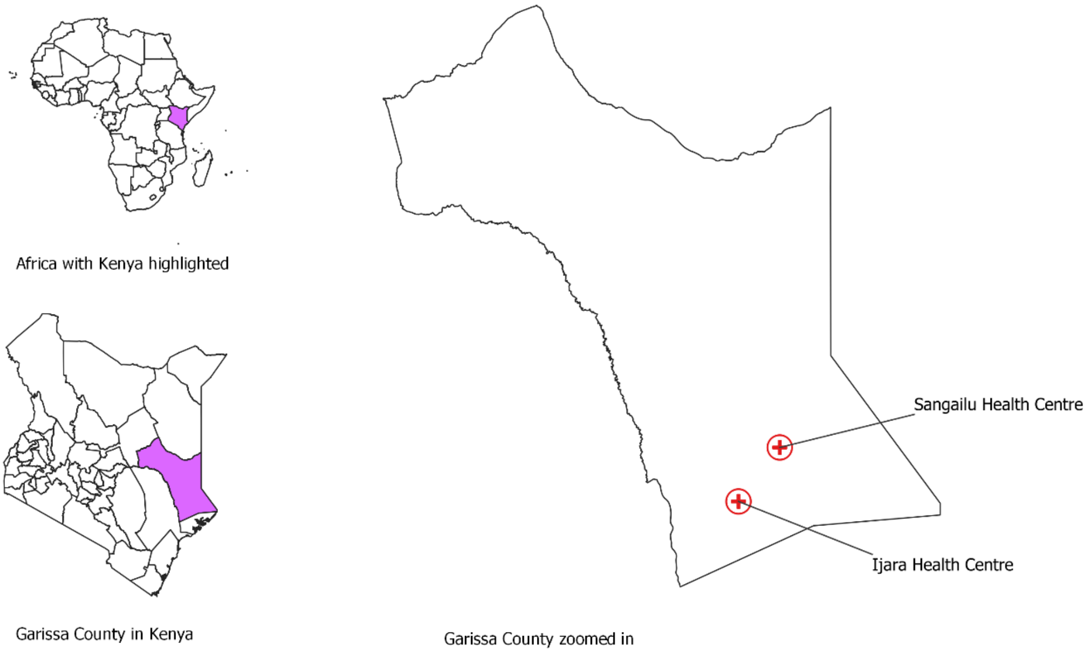

Supplementary figure 1: A map of the two health centres used to recruit febrile patients in Garissa County.

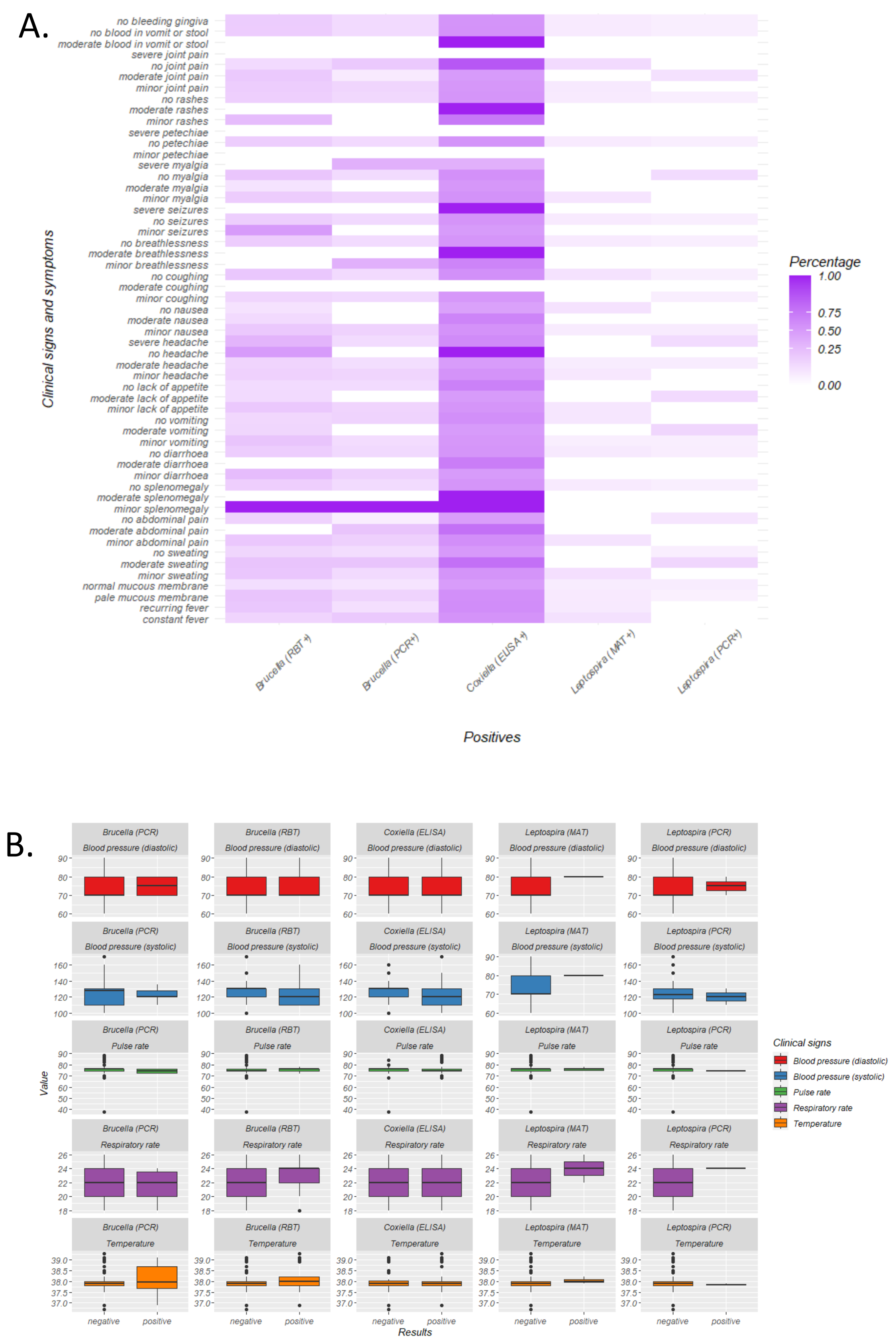

Supplementary figure 2: Clinical findings associated with febrile patients that tested sero- and PCR-positive for *Brucella* spp., *Coxiella burnetii*, and *Leptospira* spp. **A.** represents arcsine-transformed proportions of patients positive for the three zoonoses with all categorical clinical signs and symptoms, and **B.** the values observed for clinical findings (continuous variables) in positive versus negative patients.

Supplementary tables

Supplementary table 1: A distribution of proportional morbidity rates (PMr) of the three selected bacterial zoonoses with population characteristics and risk factors for exposure.

		Brucella spp.			*Coxiella burnetii*			*Leptospira* spp.		
**Variable**	**Category**	**Pos/Total**	**% (95% CI)**	χ^2	**Pos/Total**	**% (95% CI)**	χ^2	**Pos/Total**	**% (95% CI)**	χ^2
**Total**		**30/216**	13.9 (9.6-19.2)		**99/216**	45.8 (39.1-52.7)		**8/216**	3.7 (1.6-7.2)	
Gender	Female	18/150	12.0 (7.3-18.3)	0.319	71/150	47.3 (39.1-55.6)	0.604	4/150	2.7 (0.7-6.7)	0.409
	Male	12/66	18.2 (9.8-29.6)		28/66	42.4 (30.3-55.2)		4/66	6.1 (1.7-14.8)	
Health facility	Ijara	10/98	10.2 (5.0-18.0)	0.219	36/98	36.7 (27.2-47.1)	0.021	3/98	3.1 (0.6-8.7)	0.925
	Sangailu	20/118	16.9 (10.7-25.0)		63/118	53.4 (44.0-62.6)		5/118	4.2 (1.4-9.6)	
Age categories	5 to 17	11/49	22.4 (11.8-36.6)	0.138	30/49	61.2 (46.2-74.8)	0.108	2/49	4.1 (0.5-14.0)	0.985
	18 to 24	6/45	13.3 (5.1-26.8)		19/45	42.2 (27.7-57.8)		2/45	4.4 (0.5-15.1)	
	25 to 34	9/63	14.3 (6.7-25.4)		26/63	41.3 (29.0-54.4)		2/63	3.2 (0.4-11.0)	
	35 to 80	4/59	6.8 (1.9-16.5)		24/59	40.7 (28.1-54.3)		2/59	3.4 (0.4-11.7)	
Highest education	Never attended school	18/148	12.2 (7.4-18.5)	0.557	67/148	45.3 (37.1-53.7)	0.829	5/148	3.4 (1.1-7.7)	0.871
	Primary incomplete	9/51	17.6 (8.4-30.9)		25/51	49.0 (34.8-63.4)		2/51	3.9 (0.5-13.5)	
	Primary complete/Secondary	3/17	17.6 (3.8-43.4)		7/17	41.2 (18.4-67.1)		1/17	5.9 (0.1-28.7)	
Source of income	Business/Farming	2/32	6.3 (0.8-20.8)	0.202	12/32	37.5 (21.1-56.3)	0.524	0/32	0.0 (0.0-10.9)	0.296
	None	27/167	16.2 (10.9-22.6)		78/167	46.7 (40.0-54.6)		8/167	4.8 (2.1-9.2)	
	Others *	1/17	5.9 (0.1-28.7)		9/17	52.9 (27.8-77.0)		0/17	0.0 (0.0-19.5)	
Water sources	Dams and springs	13/61	21.3 (11.9-33.7)	0.141	34/61	55.7 (42.4-68.5)	0.013	3/61	4.9 (1.0-13.7)	0.839
	Unprotected wells	10/93	10.8 (5.3-18.9)		32/93	34.4 (24.9-45.0)		3/93	3.2 (0.7-9.1)	
	Others †	7/62	11.3 (4.7-21.9)		33/62	53.2 (40.1-66.0)		2/62	3.2 (0.4-11.2)	
Distance to	<7 km	11/92	12.0 (6.1-20.4)	0.611	43/92	46.7 (36.3-57.4)	0.927	4/92	4.3 (1.2-10.8)	0.946
	≥7 km	19/124	15.3 (9.5-22.9)		56/124	45.2 (36.2-54.3)		4/124	3.2 (0.8-8.1)	

Variable	Category	*Brucella* spp.			*Coxiella burnetii*			*Leptospira* spp.		
		Pos/Total	% (95% CI)	χ^2	Pos/Total	% (95% CI)	χ^2	Pos/Total	% (95% CI)	χ^2
**Total**		**30/216**	13.9 (9.6-19.2)		**99/216**	45.8 (39.1-52.7)		**8/216**	3.7 (1.6-7.2)	
healthcare facility										
Livestock enclosed	Yes	0/1	0.0 (0.0-97.5)	1.000	0/1	0.0 (0.0-97.5)	1.000	0/1	0.0 (0.0-97.5)	1.000
	No	30/215	14.0 (9.6-19.3)		99/215	46.0 (39.2-53.0)		8/215	3.7 (1.6-7.2)	
Sheep herding or direct contact ‡	Yes	24/157	15.3 (10.0-21.9)	0.802	78/157	49.7 (41.6-57.8)	0.060	7/157	4.5 (1.8-9.0)	0.889
	No	5/41	12.2 (4.1-26.2)		13/41	31.7 (18.1-48.1)		1/41	2.4 (0.1-12.9)	
Camel herding or direct contact ‡	Yes	4/16	25.0 (7.3-52.4)	0.394	7/16	43.8 (19.8-70.1)	1.000	1/16	6.3 (0.2-30.2)	1.000
	No	25/182	13.7 (9.1-19.6)		84/182	46.2 (38.8-53.7)		7/182	3.8 (1.6-7.8)	
Sheltering livestock ‡	Yes	8/65	12.3 (5.5-22.8)	0.662	27/65	41.5 (29.4-54.4)	0.471	3/65	4.6 (1.0-12.9)	1.000
	No	21/133	15.8 (10.0-23.1)		64/133	48.1 (39.4-56.9)		5/133	3.8 (1.2-8.6)	
Disposal of carcasses ‡	Yes	7/65	10.8 (4.4-20.9)	0.387	32/65	49.2 (36.6-61.9)	0.621	2/65	3.1 (0.4-10.7)	0.923
	No	22/133	16.5 (10.7-24.0)		59/133	44.4 (35.8-53.2)		6/133	4.5 (1.7-9.6)	
Butchering of livestock ‡	Yes	9/60	15.0 (7.1-26.6)	1.000	24/60	40.0 (27.6-53.5)	0.340	2/60	3.3 (0.4-11.5)	1.000
	No	20/138	14.5 (9.1-21.5)		67/138	48.6 (40.0-57.2)		6/138	4.3 (1.6-9.2)	
Milking livestock ‡	Yes	28/179	15.6 (10.7-21.8)	0.381	85/179	47.5 (40.0-55.1)	0.280	7/179	3.9 (1.6-7.9)	1.000
	No	1/19	5.3 (0.1-26.0)		6/19	31.6 (12.6-56.6)		1/19	5.3 (0.1-26)	
Drink raw sheep milk ‡	Yes	15/103	14.6 (8.4-22.9)	1.000	39/103	37.9 (28.5-48.0)	0.025	4/103	3.9 (1.1-9.6)	1.000
	No	14/95	14.7 (8.3-23.5)		52/95	54.7 (44.2-65.0)		4/95	4.2 (1.2-10.4)	

Variable	Category	*Brucella* spp.			*Coxiella burnetii*			*Leptospira* spp.		
		Pos/Total	% (95% CI)	χ^2	Pos/Total	% (95% CI)	χ^2	Pos/Total	% (95% CI)	χ^2
**Total**		**30/216**	13.9 (9.6-19.2)		**99/216**	45.8 (39.1-52.7)		**8/216**	3.7 (1.6-7.2)	
Drink raw goat milk ‡	Yes	16/125	12.8 (7.5-20.0)	0.451	51/125	40.8 (32.1-49.9)	0.079	4/125	3.2 (0.9-8)	0.681
	No	13/73	17.8 (9.8-28.5)		40/73	54.8 (42.7-66.5)		4/73	5.5 (1.5-13.4)	
Drink raw cow milk ‡	Yes	16/123	13.0 (7.6-20.3)	0.530	50/123	40.7 (31.9-49.9)	0.079	4/123	3.3 (0.9-8.1)	0.727
	No	13/75	17.3 (9.6-27.8)		41/75	54.7 (42.7-66.2)		4/75	5.3 (1.5-13.1)	
Sleep near animals ‡	Yes	8/75	10.7 (4.7-19.9)	0.303	33/75	44.0 (32.5-55.9)	0.776	2/75	2.7 (0.3-9.3)	0.693
	No	21/123	17.1 (10.9-24.9)		58/123	47.2 (38.1-56.4)		6/123	4.9 (1.8-10.3)	
Sleep outside with the herd ‡	Yes	4/46	8.7 (2.4-20.8)	0.287	23/46	50.0 (34.9-65.1)	0.646	2/46	4.3 (0.5-14.8)	1.000
	No	25/152	16.4 (10.9-23.3)		68/152	44.7 (36.7-53.0)		6/152	3.9 (1.5-8.4)	
Take animals to pasture ‡	Yes	22/142	15.5 (10.0-22.5)	0.610	70/142	49.3 (40.8-57.8)	0.201	5/142	3.5 (1.2-8)	0.792
	No	6/53	11.3 (4.3-23.0)		20/53	37.7 (24.8-52.1)		3/53	5.7 (1.2-15.7)	
Migrate with animals ‡	Yes	6/48	12.5 (4.7-25.2)	0.800	16/48	33.3 (20.4-48.4)	0.060	2/48	4.2 (0.5-14.3)	1.000
	No	22/143	15.4 (9.9-22.4)		72/143	50.3 (41.9-58.8)		6/143	4.2 (1.6-8.9)	
Brand animals ‡	Yes	3/23	13.0 (2.8-33.6)	1.000	7/23	30.4 (13.2-52.9)	0.156	0/23	0.0 (0.0-14.8)	0.652
	No	22/158	13.9 (8.9-20.3)		77/158	48.7 (40.7-56.8)		7/158	4.4 (1.8-8.9)	
Skin animals ‡	Yes	18/146	12.3 (7.5-18.8)	0.293	61/146	41.8 (33.7-50.2)	0.017	6/146	4.1 (1.5-8.7)	1.000
	No	7/33	21.2 (9.0-38.9)		22/33	66.7 (48.2-82.0)		1/33	3.0 (0.1-15.8)	
Assist with birthing of livestock ‡	Yes	1/21	4.8 (0.1-23.8)	0.319	12/21	57.1 (34.0-78.2)	0.439	0/21	0.0 (0.0-16.1)	0.687
	No	24/154	15.6 (10.2-22.3)		70/154	45.5 (37.4-53.7)		7/154	4.5 (1.8-9.1)	
Dispose of aborted	Yes	6/47	12.8 (4.8-25.7)	0.857	20/47	42.6 (28.3-57.8)	0.609	2/47	4.3 (0.5-14.5)	1.000
	No	19/124	15.3 (9.5-22.9)		60/124	48.4 (39.3-57.5)		5/124	4.0 (1.3-9.2)	

Variable	Category	*Brucella* spp.			*Coxiella burnetii*			*Leptospira* spp.		
		Pos/Total	% (95% CI)	χ^2	Pos/Total	% (95% CI)	χ^2	Pos/Total	% (95% CI)	χ^2
**Total**		**30/216**	13.9 (9.6-19.2)		**99/216**	45.8 (39.1-52.7)		**8/216**	3.7 (1.6-7.2)	
animal foetuses ‡										
Take care of sick animals ‡	Yes	4/40	10.0 (2.8-23.7)	0.389	20/40	50.0 (33.8-66.2)	0.683	3/40	7.5 (1.6-20.4)	0.496
	No	21/121	17.4 (11.1-25.3)		54/121	44.6 (35.6-53.9)		4/121	3.3 (0.9-8.2)	
Take animals for veterinary services ‡	Yes	5/20	25.0 (8.7-49.1)	0.373	12/20	60.0 (36.1-80.9)	0.293	2/20	10.0 (1.2-31.7)	0.470
	No	20/139	14.4 (9.0-21.3)		62/139	44.6 (36.2-53.3)		5/139	3.6 (1.2-8.2)	

CI: confidence interval; PMr means the seroprevalence in diseased populations

‡ Due to missing data, the totals in these variables varied.

Supplementary table 2: A summary of sample reads before and after quality trimming using *fastp* for metagenomic sequencing.

Patient	Total reads before trimming	Total bases before trimming	Total reads after trimming	Total bases after trimming	Q20+ proportion after trimming	Q30+ proportion after trimming	Duplicated proportion	Insert size peak
Patient 212	947,996	140,709,081	947,650	137,672,277	0.9	0.8	0.0	149
Patient 110	1,218,850	178,496,464	1,218,584	176,206,044	0.9	0.8	0.0	149
Patient 118	576,960	79,621,325	576,706	78,849,133	0.9	0.8	0.0	149
Patient 142	28,248	3,715,441	28,232	3,676,689	0.9	0.8	0.0	149
Patient 193	1,021,714	144,579,703	1,021,286	142,572,444	0.9	0.8	0.0	149
Patient 199	1,113,132	163,732,240	1,112,840	160,783,805	0.9	0.8	0.0	149
Patient 203	838,840	115,623,025	838,426	114,200,216	0.9	0.8	0.0	149
Patient 201	765,176	105,580,135	764,900	104,799,289	0.9	0.8	0.0	149
Patient 205	733,184	98,659,301	732,804	97,730,130	0.9	0.8	0.0	149
Patient 207	783,736	110,119,788	783,410	108,708,852	0.9	0.8	0.0	149
Patient 2	756,702	105,708,994	756,382	104,624,077	0.9	0.8	0.0	149
Patient 3	864,064	117,167,012	863,720	116,123,506	0.9	0.8	0.0	149
NEC	2072	302,698	2072	302,698	0.9	0.9	0.0	219

NEC: Negative Extraction Control
